# Machine learning methods for functional recovery prediction and prognosis in post-stroke rehabilitation: a systematic review

**DOI:** 10.1186/s12984-022-01032-4

**Published:** 2022-06-03

**Authors:** Silvia Campagnini, Chiara Arienti, Michele Patrini, Piergiuseppe Liuzzi, Andrea Mannini, Maria Chiara Carrozza

**Affiliations:** 1 IRCCS Fondazione Don Carlo Gnocchi Onlus, Via di Scandicci 269, 50143 Firenze, Italy; 2 Scuola Superiore Sant’Anna, Viale Rinaldo Piaggio 34, 56025 Pontedera, Italy

**Keywords:** Automated pattern recognition, Clinical, Efficacy treatment, Machine learning, Prognosis, Regression analysis, Rehabilitation, Rehabilitation outcome, Stroke

## Abstract

**Background:**

Rehabilitation medicine is facing a new development phase thanks to a recent wave of rigorous clinical trials aimed at improving the scientific evidence of protocols. This phenomenon, combined with new trends in personalised medical therapies, is expected to change clinical practice dramatically. The emerging field of Rehabilomics is only possible if methodologies are based on biomedical data collection and analysis. In this framework, the objective of this work is to develop a systematic review of machine learning algorithms as solutions to predict motor functional recovery of post-stroke patients after treatment.

**Methods:**

We conducted a comprehensive search of five electronic databases using the Patient, Intervention, Comparison and Outcome (PICO) format. We extracted health conditions, population characteristics, outcome assessed, the method for feature extraction and selection, the algorithm used, and the validation approach. The methodological quality of included studies was assessed using the prediction model risk of bias assessment tool (PROBAST). A qualitative description of the characteristics of the included studies as well as a narrative data synthesis was performed.

**Results:**

A total of 19 primary studies were included. The predictors most frequently used belonged to the areas of demographic characteristics and stroke assessment through clinical examination. Regarding the methods, linear and logistic regressions were the most frequently used and cross-validation was the preferred validation approach.

**Conclusions:**

We identified several methodological limitations: small sample sizes, a limited number of external validation approaches, and high heterogeneity among input and output variables. Although these elements prevented a quantitative comparison across models, we defined the most frequently used models given a specific outcome, providing useful indications for the application of more complex machine learning algorithms in rehabilitation medicine.

**Supplementary Information:**

The online version contains supplementary material available at 10.1186/s12984-022-01032-4.

## Background

Vascular problems in nature are the leading cause of death, and stroke is ranked second in worldwide mortality [1]. It accounted for 5.5 million deaths in 2006 [2]. Indeed, for survivors, the burden of stroke is producing an increase in the number of disability-adjusted living years (DALYs). For this reason, the ultimate challenge in stroke rehabilitation research is to improve the rehabilitation protocols by tuning them according to an optimised early outcome prognosis [3]. Therefore, advances in artificial intelligence, machine learning (ML), and more generically data-driven tools, may have a central role in rehabilitation decision-making and protocol development. ML is the methodology that provides computers with the ability to learn from experience. By designing and training algorithms able to learn decision rules from data, automatic solutions able to make predictions on new data can be exploited [4].

ML algorithms have been used often in recent years to predict clinical outcomes [5]. The recent growing interest is due to the increasing complexity and numerosity of available data sets, as well as the presence of multifactorial data with diverse origins, for which more classical methods do not allow accurate results [6, 7].

From this perspective and given the available technologies, a new concept of rehabilitation is arising, namely “Rehabilomics”. This innovative view of the rehabilitative intervention concerns a multifactorial data-driven evaluation of the patient, aiming at the identification of physiological, genetic, biochemical or metabolic biomarkers as factors concurring in the rehabilitation process. The correlation of these biomarkers with the clinical outcome that measures the recovery of the patient could lead to important information for rehabilitation treatment planning.

Considering the latest advances in ML-based predictive models could be employed to promote the development of personalised rehabilitation processes for individual recovery. This would result in a human-centred framework in which the synergy among therapies, biogenetics, imaging techniques, technological devices and data-driven tools has a key role [8].

In the literature, there has been a broad exploration of solutions for outcome prediction in medicine applications [6, 9–11], and very few of them are about ML models in stroke rehabilitation [12, 13]. Most of the reviews in this field provide only a narrative description of the studies, without providing a systematic analysis of the results. On the other hand, those prioritising the technical aspects of the models often lack a clinical contextualisation of the findings. For example, Christodoulou et al. [6], ML methods for clinical outcome prediction are compared across pathologies without providing details about the outcomes used. So, although the review was highly detailed from the technical point of view, i.e. regarding the algorithms validation approaches and performance metrics used, the clinical aspects were out of focus. We are convinced that a proper discussion of the results in light of the clinical context (i.e., pathology and measures) in which they are obtained is essential for translational applicability of the solutions developed, from research to the clinical practice.

Thus, there is an urgent need for a study able to integrate and combine clinical and engineering/technical aspects of predictive solutions used in rehabilitation. The aim of this study is to identify the predictive solutions, developed using ML or theory-based algorithms and internally or externally validated, used for functional outcome prognosis in stroke patients after a rehabilitation programme. The predictive solutions were investigated comprehensively, by evaluating their technical characteristics and performances in association with the clinical selection of input and output variables.

## Methods

### Study design

A systematic review has been performed following the Preferred Reporting Items for Systematic Reviews and Meta-Analyses (PRISMA) guidelines [14]. The protocol was registered on PROSPERO (ID CRD42020164184).

### Selection criteria

The eligibility criteria of the studies included in the review followed the Patient, Intervention, Comparison and Outcome (PICO) framework.

- Type of studies
  We searched for all types of primary studies, excluding only reviews and overviews from the search.
- Types of participants
  We included in the study all adult participants (over 18 years old) with stroke, independently of the type of stroke or the time post-onset (TPO).
- Types of intervention
  We included all the studies evaluating predictive models for outcome prognosis after rehabilitation treatment. We defined predictive models as either ML or theory-based algorithms trained on data and internally or externally validated on new data. Primary studies were excluded when the validation of the models, either internal or external, was not performed. We denoted as external the validation performed on new data, unseen from the model during the training phase and geographically and/or temporally independent from the training set. On the contrary, internal validation refers to methods involving only data from a single data acquisition campaign, eventually split into multiple subsets.
  Moreover, we considered the outcome of the model as a variable related to the motor functional status of the patient after the rehabilitation treatment, and we considered as predictors any variable related to the patients’ conditions before or during the rehabilitation. So, we included studies that evaluated the relationships between predictors and response, describing the functional recovery of the patient during the rehabilitation.
- Types of outcome
  We selected studies evaluating motor functional outcomes and excluded studies involving only cognitive or only sensory-related outcomes. Because functional measures are less influenced than cognitive ones by external factors such as social and cultural biases, we preferred to limit our analysis to them. Nevertheless, we decided not to excessively constrain the selection of the outcome, including either upper and lower limb-related outcomes. Both features describing lower and higher-level domains with respect to the International Classification of Functioning, Disability and Health (ICF) were included, e.g. body functions activities and participation. We also discarded all studies considering responses collected more than three months after the end of the rehabilitation treatment to focus on the effective impact of the rehabilitation phase on the outcome.

### Search methods for identification of studies

A systematic search was conducted in the following databases: PubMed, Web of Science, Scopus, CINAHL and the CENTRAL. The keywords used in the search string were ‘stroke’, ‘machine learning’, ‘regression analysis’, ‘automated pattern recognition’, ‘prognosis’, ‘rehabilitation outcome’, ‘clinical’, ‘efficacy treatment’ and ‘rehabilitation’. The search string was built using the PICO framework for prognostic studies [15]. Table 1 reports the search strings used in the different databases.

**Table 1:** Search string

Database	Search string
PubMed	((“machine learning”[MeSH Terms] OR “regression analysis”[MeSH Terms] OR “automated pattern recognition”[MeSH Terms]) AND (“stroke”[MeSH Terms]) AND (“rehabilitation”[MeSH Terms]) AND (“prognosis”[MeSH Terms] OR “rehabilitation outcome”[MeSH Terms] OR “clinical”[MeSH Terms] OR “efficacy treatment”[MeSH Terms])) OR ((“Machine Learning” OR “pattern recognition” OR “automated pattern recognition” OR “classif*” OR “regress*” OR “regression analysis”) AND (“stroke”) AND (“rehab*”) AND ((“pred*”) AND (“prognosis” OR “rehabilitation outcome” OR “clinical” OR “efficac*” OR “efficacy treatment” OR “treatment effect” OR “treatments effect”))) Sort by: Best Match Filters: English
Web of Science	(TS = ((“Machine Learning” OR “pattern recognition” OR “automated pattern recognition” OR “classif*” OR “regress*” OR “regression analysis”) AND (“stroke”) AND (“rehab*”) AND ((“pred*”) AND (“prognosis” OR “rehabilitation outcome” OR “clinical” OR “efficac*” OR “efficacy treatment” OR “treatment effect” OR “treatments effect”)))) AND LANGUAGE: (English)
Scopus	TITLE-ABS-KEY ((“Machine Learning” OR “pattern recognition” OR “automated pattern recognition” OR “classif*” OR “regress*” OR “regression analysis”) AND (“stroke”) AND (“rehab*” OR “rehabilitation”) AND ((“pred*”) AND (“prognosis” OR “rehabilitation outcome” OR “clinical” OR “efficac*” OR “efficacy treatment” OR “treatment effect” OR “treatments effect”))) AND (LIMIT-TO (LANGUAGE, “English”))
CENTRAL	((pred*) AND (prognosis OR “rehabilitation outcome” OR clinical OR efficac* OR “efficacy treatment” OR “treatment effect” OR “treatments effect”)) AND (“Machine Learning” OR “pattern recognition” OR “automated pattern recognition” OR classif* OR regress* OR “regression analysis”) AND (stroke) AND (rehab*)
CINAHL	((“Machine Learning” OR “pattern recognition” OR “automated pattern recognition” OR “classif*” OR “regress*” OR “regression analysis”) AND (“stroke”) AND (“rehab*”) AND ((“pred*”) AND (“prognosis” OR “rehabilitation outcome” OR “clinical” OR “efficac*” OR “efficacy treatment” OR “treatment effect” OR “treatments effect”)))

Once the results of each database search were merged, two independent reviewers (SC and MP) screened the papers, first by title and abstract, and then with the full text. A third reviewer was involved in case of disagreements (AM). During this phase, only papers in English were considered eligible for screening. The selection concerning outcomes was not applied during the search phase; it was involved in the screening phase only.

### Data collection

The CHecklist for critical Appraisal and data extraction for systematic Reviews of prediction Modelling Studies (CHARMS) was used [16]. The data extracted from the included studies concerned:

- Source of data: authors, publication year, study design and DOI.
- Participant characteristics: age, number, specifications of the stroke event both in terms of aetiology and TPO.
- Setting: monocentric or multicentric, type.
- Outcomes: type, measures used, the timing of acquisition with respect to the rehabilitation treatment.
- Predictors: type, measures used, the timing of acquisition with respect to the rehabilitation treatment, number.
- Data treatment: number of missing data and treatment of missing data.
- Methods used: features selection approach, the algorithm used, internal or external validation strategy.
- Model performances: metrics used for performance evaluation, performance reported, limitations reported.

### Assessment of risk of bias of the included studies

The Prediction model Risk Of Bias ASsessment Tool (PROBAST) was used for the assessment of the methodological quality of the included studies [17]. The PROBAST tool is helpful to evaluate both the risk of bias and applicability of the included predictive models in four domains (participants, predictors, outcome and analysis).

### Data synthesis

**Fig. 1:**
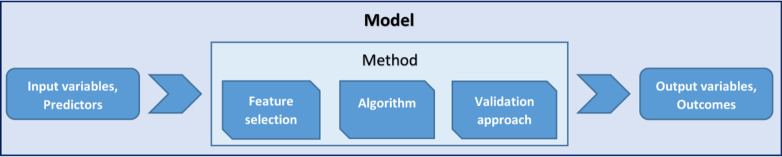
Terminology used in this review paper regarding the technical steps and parts of the models

To approach more clearly the description of the results, an illustration of the terminology we used is required (Fig. 1). The *model* is intended as the complex ensemble of predictors, computational methods and outcome variables. The term *variables* refers to both the input features (or *predictors*) and the *outcomes* of the models. Finally, *methods* addresses the computational ensemble of the feature selection process, algorithm and validation approach characterising the model.

Due to the heterogeneity of the selected populations, as well as the heterogeneity of the model characteristics (as detailed in the following sections), we decided not to perform a meta-analysis. Instead, a qualitative analysis was conducted, based on the data extracted from the systematic search.

First, a description of the population and general characteristics of the studies was generated. Then, a frequency analysis was conducted, investigating separately the variables and methods that were used. Specifically, in the analysis of the variables, the type of predictors and outcomes, the instruments used to define them, as well as the most used associations among the input and output features were investigated. All parts of the methods were analysed, that is, the algorithms for the training, the validation approach and the feature selection strategy (when used).

Given that in our work the studies could report the implementation of one or more models, the analysis was performed considering for each study the best-performing ones. More specifically*,* we selected the best models for each outcome measure (Barthel Index, speed, etc.) and type (categorical, ordinal or numerical). The performance was evaluated using the same metrics reported by the studies.

Finally, a summary description of the reviewed models was reported. Based on the results obtained in the single parts of the models applied in the different studies (methods, variables and performances analysed separately), a critical discussion of methods with respect to the predictors and outcomes was presented to show the state of the art of currently available models versus outcomes. The association among the variables (outcome measures, outcome classes and predictor classes) and the methods (validation approaches and algorithms) was additionally sustained by graphical means with alluvial charts. By reading the alluvial charts either from right to left or vice versa, it is possible to connect the information among the domains included. In particular, the thickness of the flows is giving a visual indication of the strength of the specific connection.

## Results

**Fig. 2:**
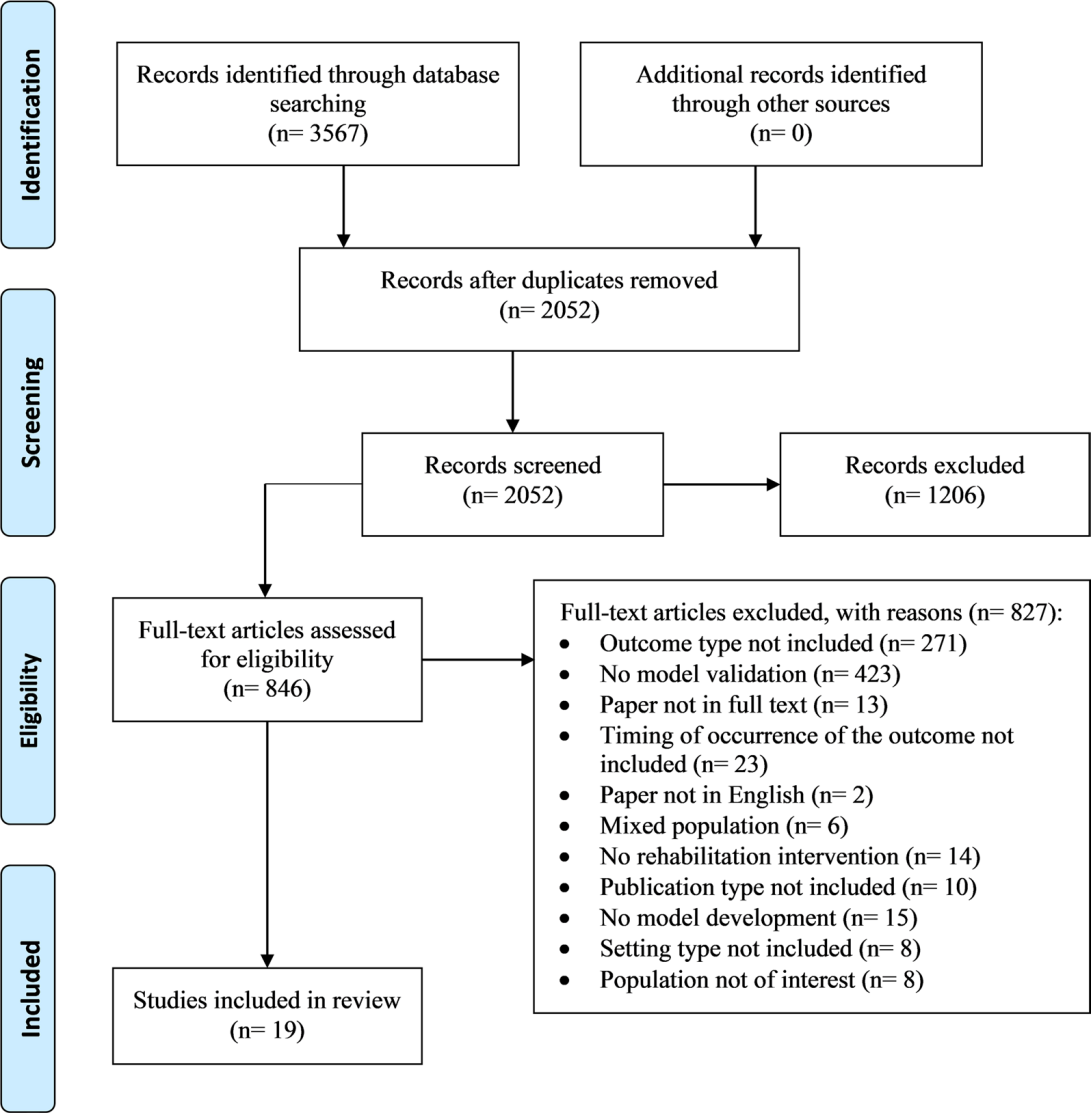
Study flow-chart. It is reported the number of papers screened and the reasons for exclusion

The electronic search resulted in 3567 papers. No additional records were identified through other sources. After removing duplicates and screening the titles and abstracts, there were 846 studies for full text screening. At the end, 19 results [18–36] were included in the study (Fig. 2). It is important to point out that the most relevant selection of the studies occurred during the full text screening rather than during the title and abstract selection phase. This is partly due to the selection criteria on the outcome and on the intervention criteria. Indeed, it was necessary to analyse the full text to ascertain the presence of a proper validation of the model, in order to assess the actual presence of a predictive model.

The criterion related to the type of intervention was the reason for the exclusion of 423 studies since the main focus of these papers was not the development and validation of predictive models, but an explorative analysis aiming at the identification of biomarkers and the investigation of their association with the outcome of interest.

In Tables 2, 3 and 4, reduced versions of the data extraction, as well as a summary of the methodological quality of the studies, are shown; the full version of the data extraction table is available in the Additional file 1. For each included primary study, a detail of the models with the best performance is provided in terms of outcome type, measure and time of acquisition, predictor type and time of acquisition, feature selection method, algorithm, validation approach and performance measure. Moreover, an indication of the total number of models investigated in the study is given. For brevity and in order to provide a weighted description of the state of the art at a study level, the characteristics of the models are given for the best-performing ones only, despite the fact that data of all the models were extracted in depth.

**Table 2:** Population characteristics. Information regarding the sample size, age, additional aetiology-related inclusion criteria, and outcome type are presented

Study	Age (mean (std) or [range])	Sample size	Further inclusion criteria specifications regarding stroke pathology (time from event or aetiology)	Outcome
Almubark et al.	N/R	45	Event happened more than 6 months before the study	Upper extremity home use
Bates et al.	70.4 (11.47)	4020	N/A	Physical grade achievement
Berlowitz et al.	67.7 (11.1)	2402	N/A	Functional outcome
Bland et al.	[21–93]	269	N/A	Walking ability
Cheng et al.	N/R	82	Ischemic	Recovery
Li et al.	65.6 (12.31)	271	First-ever ischemic	Functional status
De Marchis et al.	[60–83]	1102	Acute ischemic	Unfavourable functional outcome
de Ridder et al.	PAIS: 70.1 (13.4) PRACTISE: 70.6 (13.4) PASS: 71.9 (12.5)	PAIS = training = 1227 PASS = validation = 2125 (2107) PRACTISE = validation = 1657 (1589)	Ischemic	Disability and functional outcome
George et al.	[24–84]	35	Chronic	Extent of motor recovery after constraint-induced movement therapy
König et al.	Original: 68.1 (12.7) VISTA: 68.8 (12.3)	Original = 1754 VISTA = 5048	Acute ischemic	Functional independence
Kuceyeski et al.	72.0 (12.0)	41	Ischemic	Clinical performance
Abdel Majeed et al.	Control arm: 55.54 (12.63) Treatment arm: 55.23 (9.11)	26	Chronic	Change in clinical outcomes
Masiero et al.	Construction set: 69 (12) Validation set: 68 (11)	150	Recent stroke (< 8 weeks post-event)	Ambulation
Mostafavi et al.	N/R	126		Assessment of impairment
Sale et al.	N/R	55	Subacute (15 ± 10 days from injury)	Motor improvement
Scrutinio, Lanzillo, et al.	Derivation set: 72 (12) Validation set: 70 (12)	1592	N/A	Functional status
Scrutinio, Guida, et al.	[65–80]	951	30 days from stroke occurrence	Treatment failure
Sonoda et al.	Prediction group: 63.4 Validation group: 65.2	131	N/A	Stroke outcome
Zariffa et al.	[60–73]	9	Chronic	Measure of upper-limb function

*N/R* information should be specified but it is not reported in the paper, *N/A* information not applicable to the specific paper

**Table 3:** PROBAST

Criteria	Specification of the review question
Step 1: Specify your systematic review question	
Intended use of the model:	Prediction of functional outcome after rehabilitation treatment of post-stroke patients
Participants:	Adults post-stroke participants selected independently on the timing of the event or type of stroke
Predictors:	Any kind of predictor was included, more specifically any type included in the following categories of stroke assessment: biomechanical assessment, functional assessment, demographic characteristics, medical history, stroke assessment and neurological assessment. The selected predictors are related to the admission or recovery phase only, excluding predictors variables collected at discharge
Outcome:	Any kind of functional outcome, not exclusively cognitive or sensory-related was selected

Study	Outcome	Type of prediction study
Step 2: Classify the type of prediction model evaluation		
Almubark et al.	Upper extremity home use	Development only
Bates et al.	Physical grade achievement	Development only
Berlowitz et al.	Functional outcome	Development only
Bland et al.	Walking ability	Development only
Cheng et al.	Recovery	Development only
Li et al.	Functional status	Development only
De Marchis et al.	Unfavourable functional outcome	Development and validation
De Ridder et al.	Disability and functional outcome	Development and validation
George et al.	Extent of motor recovery after constraint-induced movement therapy	Development only
König et al.	Functional independence	Development and validation
Kuceyeski et al.	Clinical performance	Development only
Abdel Majeed et al.	Change in clinical outcomes	Development only
Masiero et al.	Ambulation	Development only
Mostafavi et al.	Assessment of impairment	Development only
Sale et al.	Motor improvement	Development only
Scrutinio, Lanzillo, et al.	Functional status	Development only
Scrutinio, Guida, et al.	Treatment failure	Development only
Sonoda et al.	Stroke outcome	Development only
Zariffa et al.	Measure of upper-limb function	Development only

Domain	Risk of bias (number of models)		Applicability (number of models)	
	Dev	Val	Dev	Val
Step 3: Assess risk of bias and applicability				
Participants	High = 0 Unclear = 0 Low = 174	High = 0 Unclear = 0 Low = 174	High = 0 Unclear = 0 Low = 174	High = 0 Unclear = 0 Low = 174
Predictors	High = 1 Unclear = 0 Low = 173	High = 1 Unclear = 0 Low = 173	High = 1 Unclear = 0 Low = 173	High = 1 Unclear = 0 Low = 173
Outcome	High = 24 Unclear = 120 Low = 30	High = 24 Unclear = 120 Low = 30	High = 24 Unclear = 119 Low = 31	High = 24 Unclear = 119 Low = 31
Analysis	High = 77 Unclear = 8 Low = 89			
Overall	High = 85 Unclear = 67 Low = 22		High = 35 Unclear = 110 Low = 29	

A short table containing the details on the four steps of the evaluation is reported

**Table 4:** Data extraction table

Study	Number of models in the study	Outcomes	Outcome measure (type of outcome, ICF classification)	Outcome at discharge? Yes/no	Predictors (number)	Timing of the measurement	Methods for features selection	Algorithm of the best performing model	Validation approach	Measures and methods used for the description of model performance
Almubark et al.	102	Upper extremity use at home	MAL ratio (dichotomous variable, d5d6)	N/R	Trunk compensation, ARAT (3)	N/R	N/A	RF after PCA	Leave-One-Subject-Out	Classification accuracy 93.33%
		Upper extremity use at home	Accel ratio (dichotomous variable, b7)					KNN		Classification accuracy 86.66%
Bates et al.	1	Physical grade achievement	FIM (numeric variable, d2d3d4d5d7)	Yes	Anagrafic data, clinical data, comorbidities data, acute procedures (38)	N/R	Unadjusted bivariate logistic analyses _ features selected are with p < 0.2	LogR	60% -40% split	ROC area on the derivation set = 0.84 ROC area on the validation set = 0.83 + Hosmer–Lemeshow test at p = 0.93 not significant on the derivation cohort
Berlowitz et al.	4	Functional outcome	FIM change (numeric variable, d2d3d4d5d7)	Yes	Age, gender (2)	N/R	N/A	LR	Bootstrap method (1000 samples)	R^2 = 0.75
Bland et al.	2	Walking ability	10 m walking speed (dichotomous variable, b7)	Yes	Motricity Index, somatosensation of the dorsum of the foot, Modified Ashworth Scale for plantar flexors, FIM walk item, Berg Balance Scale, 10-m walk speed, age, TPO (8)	Admission	Pearson product-moment correla_ tion	LogR	110 -159 samples split	Sensitivity (0.94), specificity (0.65), OR (32), positive and negative predictive values (0.70, 0.93)
			10 m walking speed (numeric variable, b7)					LR		Sensitivity (0.94), specificity (0.65), OR (32), positive and negative predictive values (0.70, 0.93)
Cheng et al.	3	Recovery	MRS (dichotomous variable)	No, at 3 months	Gender, hypertension, heart disease, diabetes, previous stroke with yes or no nodes, age, OTT, NIHSS (8)	N/R	N/A	NN	80%—20% split	ROC curve = 0.969,sensitivity = 0.9444,specificity = 0.9565,accuracy = 0.9512
De Marchis et al.	2	Unfavourable functional outcome	MRS (dichotomous variable, d2d4)	No, at 3 months	Age, NIHSS score, thrombolysis, log10-transformed copeptin levels (4)	Admission	Chosen variables that were independently associated with 3-month functional outcome in the dev and val cohorts	LogR	Model trained on COSMOS dataset (319) and tenfold CV; Ex. validated on CoRisk dataset (783)	Brier score + AUC (0.819) + NRI = continuous net reclassification index (0.46)
De Ridder et al.	7	Functional outcome	MRS (dichotomous variable, d2d4)	No, at 3 months	Gender; age; NIHSS, Diabetes, previous stroke atrial fibrillation and hypertension (7)	N/R	Selected variables that were clinically relevant and/or previously reported to predict outcome in the literature	LR	Model trained on PAIS dataset (1227) and ex. validated on PASS dataset (2107)	AUC = 0.81
George et al.	6	Extent of motor recovery after constraint-induced movement therapy	WMFT (dichotomous variable, d2d4)	Yes	Side of motor impairment, motor predictors: each of the 15 WMFT natural-log-transformed item times; Sensory-motor predictors: BKT score, TM for the affected side (18)	N/R	All possible combinations of 18 inputs, a total of 262,125 combinations, were generated	NN	35 different splits at different random ratios (RTT)	Accuracy = 100%
König et al.	1	Functional independence	BI (dichotomous variable, d2d4d5)	No, at 3 months	Single items as well as the overall score of the NIHSS (16)	N/R	Systematic literature search	LogR	Model trained on original dataset (1754); ex. validated on VISTA dataset (5048)	AUC = 72.9%
Sonoda et al.	2	Stroke outcome	Motor FIM (numerical variable, d2d4d5)	Yes	Total cognitive subscore of the FIM, age, days from stroke onset to dmission, motor-FIM (4)	Admission	N/A	LR	87 -44 samples split	Correlation coefficients = 0.93
Kuceyeski et al.	7	Clinical performance	Motor FIM (numerical variable, d2d4d5)	N/R	Right inferior occipital and calcarine areas (N/R)	N/R	Jackknife CV	LR	Bootstrap	Akaike Information Criterion (AIC) and R^2 = 0.45 (0.08)
			FIM (numerical variable, d2d3d4d5d7)							Akaike Information Criterion (AIC) and R^2 = 0.37 (0.08)
			MI (numerical variable, b7)							Akaike Information Criterion (AIC) and R^2 = 0.54 (0.14)
Li et al.	2	Functional status	BI (numerical variable, d2d4d5)	Yes	Demographic information (age, sex and smoking habit), medical history (hypertension, diabetes mellitus, atrial fibrillation and hypercholesterolemia), evaluation at initial admission in the emergency department (blood glucose, blood pressure, laboratory data and the stroke severity) (N/R)	Admission	N/A	LR	CV (90–10% _ split)	R^2 adjusted = 0.573
Scrutinio, Lanzillo, et al.	2	Functional status	FIS (dichotomous variable, d2d4d5)	Yes	Age, sex, marital staus, employment status, hypertension, diabetes mellitus, COPD, coronary heart disease, atrial fibrillation, TPO, aetiology, side of impairment, aphasia, unilateral neglect, M-FIM, cognitive FIM, blood urea nitrogen, estimated glomerular filtration rate, hemoglobin (19)	Admission	Forward stepwise selection approach with P < 0.05	LogR	717–875 samples split	AUC (0.913), Hosmer–Lemeshow test ( 1.20 (P = 0.754)) and calibration plots
			Motor FIM (dichotomous variable, d2d4d5)							AUC (0.883), Hosmer–Lemeshow test ( 4.12 (P = 0.249)) and calibration plots
Mostafavi et al.	12	Assessment of impairment	MAS (numerical variable, b7)	Yes	postural hand speed; reaction and its timing; initial movement direction error/ratio, hand speed ratio; number of speed peaks, speed ranges; movement time, hand path length, and maximum hand speed trial-to-trial variability of the active hand; contraction/expansion of the overall spatial area of the active hand relative to the passive hand; systematic shift between the passive and active hand (8)	During every session, they are instrumental attributes	N/A	PCI	tenfold CV, repeated 100 times + external valiudation	R-value, RMSE, NRMSE (0.054, 0.405, 31.2)
Masiero et al. [29]	1	Ambulation	FAC (dichotomous variable, d4)	Yes	Age, gender, arterial hypertension, hypolipoproteinaemia, diabetes, event date and aetiology, paralysed side length of hospital stay, up MI and low MI, TCT, FIM and mot FIM (12)	Admission	N/R	LogR	100–50 samples split	ROC curves (ROC area = 0.94, CI 95%: 0.86–0.96, p < 0.0001), with sensitivity of 86.5% (CI 95%: 77–96%) and specificity of 95.5% (CI 95%: 75–95%))
Abdel Majeed et al.	8	Change in clinical outcomes	FM change (numerical variable, b2b7)	Yes	Demographic/physiological characteristics descriptive statistics of movement (51)	Demogr. and physiol. at baseline, movement features	Random forests with 100 repeats of fourfold CV	LR	CV	RMSE and R^2 < 2.24%
Scrutinio, Guida, et al.	1	Treatment failure	FIM-M (dichotomous variable, d2d4d5)	Yes	Age, sex, marital status, diabetes mellitus, TPO, stroke type, side of impairment, FIM-M and cognitive scores, neglect (10)	N/R	Backward stepwise selection (P > 0.157 for exclusion)	LogR	Resampling 200 bootstrap replications	Hosmer–Lemeshow test (7.77 (PZ.456)) and AUC (0.834)
Mostafavi et al.	12	Assessment of impairment	FIM-M (numerical variable, d2d4d5)	Yes	postural hand speed; reaction and its timing; initial movement direction error/ratio, hand speed ratio; number of speed peaks, speed ranges; movement time, hand path length, and maximum hand speed trial-to-trial variability of the active hand; contraction/expansion of the overall spatial area of the active hand relative to the passive hand; systematic shift between the passive and active hand (8)	During every session, they are instrumental attributes	N/A	PCI	Tenfold CV, repeated 100 times	R-value, RMSE, NRMSE (0.562, 16.6, 21.7)
			FIM (numerical variable, d2d3d4d5d7)							R-value, RMSE, NRMSE (0.596, 16.8, 20.5)
			Purdue Pegboard score (numerical variable, d2d4)							R-value, RMSE, NRMSE (0.483, 4.1, 14.1)
Abdel Majeed et al.	8	Change in clinical outcomes	WMFT change (numerical variable, d2d4)	Yes	Demographic/physiological characteristics descriptive statistics of movement (51)	Demogr. and physiol. at baseline, movement features	Random forests with 100 repeats of fourfold CV	LR	CV	RMSE and R^2 < 4.68%
Sale et al.	9	Motor improvement	FIM-M (numerical variable, d2d4d5)	Yes	Age, gender, aetiology, first event, recombinant tissue plasminogen activator, BI, FIM motor impairment, dysphagia, tracheostomy, neuropsychological impairment, speech impairment, presence of nasogastric feeding tube, length of stay (14)	T0 = admission T1 = discharge	Mutual Information (MI) criterion	SVM	20 rep. of hold-out approach with 70%—30% split + nested fivefold CV on the training set	Correlation, RMSE and MADP (0.76, 16.32, 26.79%)
			FIM (numerical variable, d2d3d4d5d7)							Correlation, RMSE and MADP (0.79, 18.78, 18.88%)
			BI (numerical variable, d2d4d5)							Correlation, RMSE and MADP (0.75, 22.6, 83.96%)
Zariffa et al.	2	Measure of upper-limb function	FMA (numerical variable, b2b7)	Yes	Mean velocity, peak velocity, RMS jerk, mean-rectified jerk, number of peaks, path smoothness, speed smoothness, SPARC, passive ROMs, passive ROM Area, Active ROMs, Active ROM Area (14)	During 76 assessments	Exhaustive search of all the combinations of the 14 features	LR	Leave-one-subject-out	R^2 = 0.4390, SRD = 1.4621
			ARAT (numerical variable, b7)							R^2 = 0.4246, SRD = 2.6803

A short description of the methods, predictor, outcomes, and the total number of models performed is presented

*N/R* information should be specified but it is not reported in the paper, *N/A* information not applicable to the specific paper, *ARAT* Action Research Arm Test, *BI* Barthel Index, *FAC* Functional Ambulation Categories, *FIM* Functional Independence Measure, *FIM-M* Functional Independence Measure-Motor, *FIS* Fatigue Impact Scale, *FM* Fugl-Meyer, *FMA* Fugl-Meyer Assessment, *MAL* Motor Activity Log, *MAS* Modified Ashworth Scale, *MI* Motricity Index, *MRS* Modified Rankin Scale, *SDMT* Symbol Digit Modalities Test, *WMFT* Wolf Motor Function Test, *ANN* Artificial Neural Networks, *FOS* Fast Orthogonal Search, *kNN* k-Nearest Neighbours, *LR* Linear Regression, *LogR* Logistic Regression, *PCI* Parallel Cascade Identification, *RF* Random Forest, *SVM* Support Vector Machine, *AUC* Area Under the Curve, *MADP* Mean Absolute Deviation Percentage, *NRI* Net Reclassification Index, *NRMSE* Normalized Root Mean Square Error, *RMSE* Root Mean Square Error, *SRD* smallest real difference, *CV* cross-validation

### Study characteristics

We included 19 trials involving a total number of 23118 participants both for model development and validation. Eight of the included trials are multicentric studies [20, 22–24, 26, 30, 32, 33] and four of the studies with the largest sample sizes relied on shared digital databases and infrastructure for data collection [20, 21, 24, 26].

Regarding the participants, the mean age ranges from 55 to 72 years. For what concerns specific inclusion criteria related to the pathology, six studies reported a focus on ischemic stroke patients [23, 24, 26–28, 36], four studies included only stroke patients in the chronic phase (TPO > one month) [18, 19, 25], one in the subacute (2 weeks < TPO < 1 month) phase [31] and two studies included only stroke patients in the acute phase (TPO < two weeks) [23, 26]. More detailed information about the populations included in the studies is reported in Table 2.

As reported in the inclusion criteria related to the intervention, all included studies investigated predictive models for functional outcome prediction, thus, after its training, the validation of the model (either internal or external) was studied. The PROBAST tool identified only three papers reporting in the same article the external validation, i.e. performing the validation on new data independent from the training dataset content and unseen from the model [23, 24, 26]. Conversely, the remaining 16 focussed on the development only, indicating, according to the instructions of the PROBAST tool, the presence of training and internal validation of the model (Table 3).

The 19 included primary studies investigated a total of 174 different models, with studies reporting only one model, up to 102 within the same paper [19]. More in detail, 4 papers reported in the study the investigation of one model only [20, 26, 29, 32], 5 papers included in the study multiple models comparing only different outcomes or outcome types [22, 28, 30, 33, 34], whilst the remaining 10 performed multiple comparisons among outcomes, algorithms or predictors subsets. The performances of the best performing models, given the same outcome measure and type, were evaluated using the metrics reported by the authors. In presence of equally performing models, those conducted with simpler methods or on larger sample sizes were selected. As a result, 31 models were obtained, as reported in Table 4.

### Risk of bias of the included studies

Differently from what is reported in Table 4 and the results, in which only the best performing models are presented, the risk of bias analysis, was executed for every model included in the review (Additional file 1), and the overall results were determined by the evaluation given in the four domains (participants, predictors, outcome and analysis). In these analyses, with the term bias, we refer to the methodological bias caused by an imprecise reporting of the results and more generically of the experimental process.

Overall, there are 22, 67 and 85 models, respectively, rated with a ‘Low’, ‘Unclear’ and ‘High’ risk of bias, and 29, 110 and 35 models, respectively, with a ‘Low’, ‘Unclear’ and ‘High’ applicability concern.

#### Participants

The risk of bias evaluation related to the participants’ section is common for each model belonging to the same study because all the models belonging to the same study share the same population and sample size. Moreover, the ratings on the development and validation set columns are equal for this section, because the majority of the models did not rely on external datasets; for those that did, the populations did not show differences regarding the data source and inclusion criteria.

The data source as well as the inclusion criteria of the participants were always declared; thus, all the reviewed models were evaluated with a low risk of bias and a low concern for applicability.

#### Predictors

In the analysis of the predictor section, there was a low risk of bias and applicability concern for most of the models. Only one model was evaluated as ‘High’ risk due to a lack of information about the predictors used in the regression method [36].

#### Outcome

The risk of bias analysis for the outcome highlighted 31 models with a ‘Low’ rating, 120 with an ‘Unclear’ rating and 23 with a ‘High’ rating. All the models with an ‘Unclear’ or a ‘High’ evaluation had a negative or unknown answer to the question ‘Was the time interval between predictor assessment and outcome determination appropriate?’ In fact, although in these articles it was evident that rehabilitation treatment occurred between the assessment of predictors and the outcome determination, the exact timings were not clearly stated. Additionally, the models with a ‘High’ risk of bias were characterised by an unclear or inappropriate outcome definition and determination with respect to predictor knowledge.

##### Analysis

In the analysis assessment, 89 models had a ‘Low’ risk of bias, 8 had an ‘Unclear’ rating and the remaining 77 had a ‘High’ rating. The main factors affecting a ‘High’ risk of bias are the approaches for handling missing data, the awareness of overfitting during the description of the model performances and the presence (or lack thereof) of sufficient data-set numerosity, given the number of predictors. In particular, only 36 models accounted for overfitting within the paper; almost half of the models (83) from 8 different studies appeared to have insufficient participants, and only 3 of these studies reported this limitation in the results [18, 27, 34].

### Description of the input and output variables

The description of both outcomes and predictors was reported in terms of the measurement used for their definition, type of variable (categorical, ordinal or scale), the timing of acquisition (when specified by the article) and the number of variables used in the case of predictors (Additional file 1). Specifically, only results of the best models from each included study were retained within analyses.

For what concerns the treatment of missing data within the variables considered, only three papers [24, 33, 34] reported the number of patients with missing values, one of which, however, did not report the way these missing values were handled [33]. Conversely, six papers reported among the methods the techniques used for handling missing data, without explicitly specifying the number [19–22, 26, 28]. The methods mostly used were statistical imputation of missing data (mostly through median values) or sample deletion.

As previously stated, the aim of this review is to investigate the prediction of the clinical outcome after the effect of the rehabilitation treatment. Thus, to reduce the possible influence of intermediate events on the selected outcome, we constrained the upper bound of 3 months on the timing of acquisition of the outcomes. Using this approach, the majority of the models [22 in total] focussed on outcomes at discharge, 4 chose outcomes at 3-month follow-up and 5 did not specify the exact timing. For the predictors, the timing of the acquisition, i.e. the timing in which the variable is collected, was not specified in most of the models (a total of 14). In those in which it was reported, the timing was indicated at admission in 6 models, both baseline and discharge for 3 models [31] and within the rehabilitation treatment itself in the case of the remaining 8 models [18, 30, 34], in which the predictors were features deriving from instrumental data.

To be concise and to ease the performance comparison across models, both predictors and outcomes were categorised. Regarding the outcomes, the categorisation was performed using the International Classification of Functioning, Disability and Health (ICF) [37] on outcome measures. At first, each outcome measure was assigned with the corresponding detailed ICF classification (Table 4) then, for analyses, the outcomes were distinguished among those related to body functions and those related to activities and participation.

It emerged that in some cases the same clinical scale used for the outcome definition was the expression of different outcome types, highlighting a strong heterogeneity in the use of clinical tools for functional assessment in rehabilitation.

The outcome measures resulted to be associated, for the majority of the models (23 out of 31), with activities and participation, whilst a way smaller number of models [8] attempted the prediction of outcomes related to body functions.

For what concerns the predictors, the categorisation could not be performed on the ICF model, since most of the paper did not provide the exact measures describing the features; thus, a different kind of grouping was performed. At first, the classes were blindly identified trying to address in the most complete way the stroke patients’ assessment; then, each group was populated for every model included. The proposed classes were the following (in brackets some examples for each class are presented):

- Demographic characteristics (age, gender, marital status, employment status…).
- Medical history (presence of hypertension, presence of diabetes mellitus, presence of chronic obstructive pulmonary disease, presence of chronic heart disease…).
- Stroke assessment through clinical evaluation (length of stay, presence of dysphagia, presence of nasogastric tube, presence of tracheostomy…).
- Stroke assessment through laboratory analysis (presence of recombinant tissue plasminogen activator, blood urea nitrogen, haemoglobin…).
- Stroke assessment through imaging (area of the left supramarginal gyri obtained by MRI, area of the right thalamus obtained by MRI, area of the left superior parietal regions obtained by MRI).
- Functional assessment (Motricity Index score, Modified Barthel Index score, Berg Balance Scale score…).
- Neurological assessment through clinical examination (side of the impairment, type of stroke, TPO…).
- Neurological assessment through instrumental examination (not reported).
- Biomechanical assessment through clinical examination (10 m walking test speed).
- Biomechanical assessment through instrumental examination (mean velocity from robotics assessment, peak velocity from robotics assessment, passive range of motion from robotics assessment, active range of motion from robotics assessment…).

**Fig. 3:**
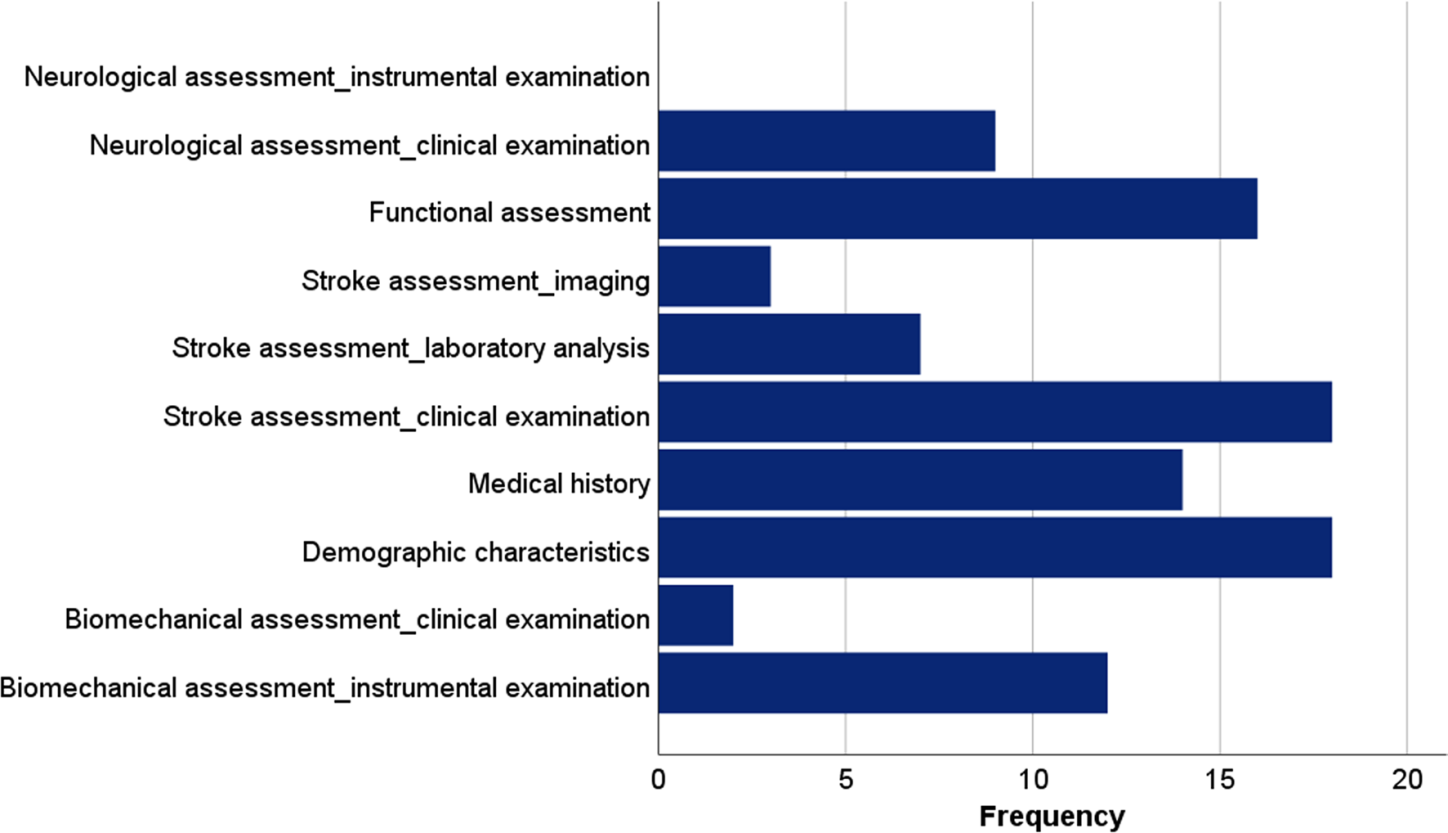
Frequencies of the predictor classes among models (N = 31)

Figure 3 presents the histogram with the relative frequencies of these classes in the models. The predictor classes were not mutually exclusive, as models usually included features of different nature (up to six different classes of features were used within the same model). In particular, 11 models retained features from 1 class only, whilst 15 models out of 31 performed the training with features belonging to more than 3 classes.

It can be noticed that due to the blinded nature of this category identification, the class neurological assessment through instrumental examination is not reported because it was never observed in the included studies. On the contrary, the two most populated predictor classes used in the models were demographic characteristics and stroke assessment through clinical examination, used in 18 out of 31 models. Interestingly, among the most populated classes of predictors, it was found that the biomechanical assessment through instrumental examination was used in 12 different models.

The number of predictors ranged between 2 and 51 features, with a mean value (SD) of 14.2 (12.8). Among the models, 17 reported a process of feature selection before the development of the model, 5 of which performed it through an exhaustive search approach. However, less than half of the models adopting an automatic strategy to reduce features (8 out of 17) provided the final number of retained predictors used for the prognosis.

Regarding the use of predictors obtained through instrumental data, the features used in the included studies were related to biomechanical assessment through instrumental examination and stroke assessment through imaging. In particular, 12 models belonging to 5 different studies [18, 19, 22, 30, 34] used kinematic data among the predictors.

### Description of the methods

**Fig. 4:**
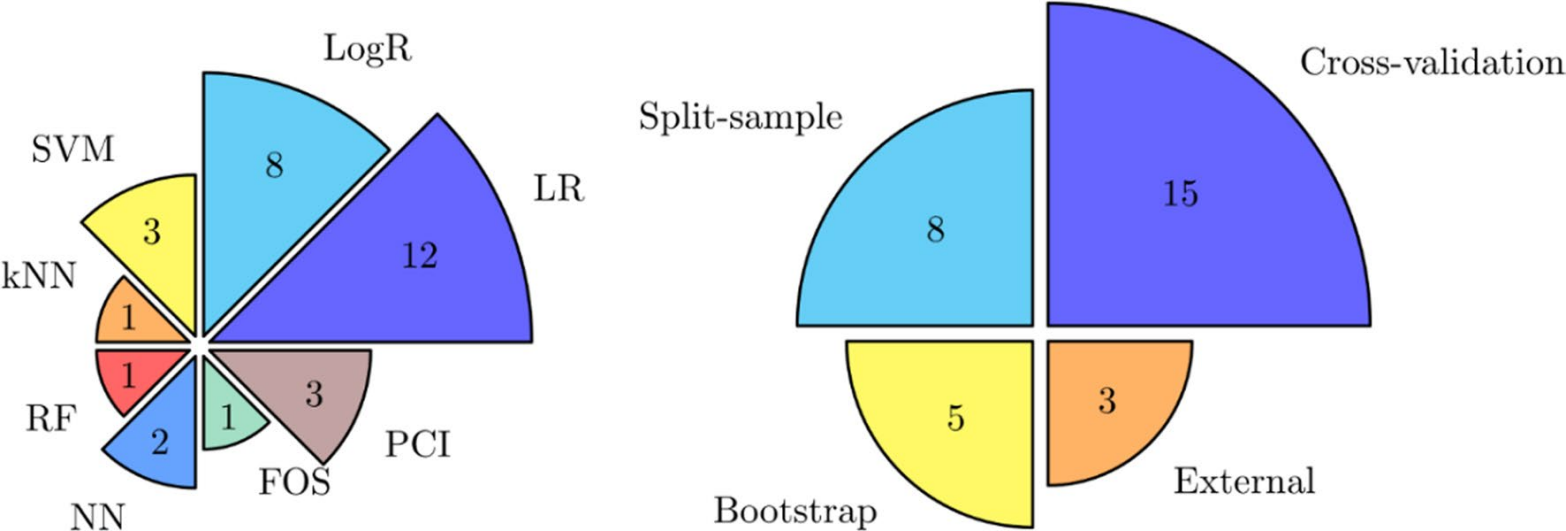
Algorithms (on the left) and validation approaches (on the right) among the best performing models (N = 31). *FOS* fast orthogonal search, *LogR* Logistic Regression, *LR* Linear Regression, *SVM* Support Vector Machines, *kNN* k-Nearest Neighbours, *RF* Random Forest, *NN* Neural Network, *PCI* Parallel Cascade Identification

The most used algorithms among models are regressions, specifically 12 models trained linear regressions and 8 models logistic ones (Fig. 4, left).

Regarding the validation process, 28 models performed internal validation, internally divided into cross-validation, split-sample and bootstrap methods, whilst only 3 models performed external validation (Fig. 4, right). More in detail, regarding the specific group in which cross-validation was performed [15], only for 10 models was clearly stated the purpose of the method, either if used for fine-tuning of hyper-parameters or performed on the same parameters for testing the generalisability of the model. In particular, Mostafavi et al. [30] and Zariffa et al. [34] reported the use of cross-validation for the optimisation of hyper-parameters, whilst Sale et al. [31] and Li et al. [28] addressed nested cross-validation for both purposes.

Cross-validation was performed also by De Marchis et al. [23], who calibrated a logistic regression with tenfold cross-validation, for the identification of the intercept, keeping fixed the regression coefficients, then an external validation was performed for the calculation of the performance metrics of the model. König et al. [26] and De Ridder et al. [24] also reported a recalibration and internal validation respectively, without stating the approach used.

### Model performances

Model performances were evaluated through several performance measures, coherently with the type of the variable used as outcome. In particular, accuracy, sensitivity, specificity, AUC, Hosmer–Lemeshow test and NRI were used for categorical outcomes, whilst the remaining R^2^, R-value, RMSE, NRMSE, MDP and SRD were used with numerical outcome variables.

The most common performance metrics for numerical and categorical outcomes were respectively the R^2^, indicating the percentage of outcome variance explained by the predictors, and the area under the curve (AUC) of the receiver operating characteristic (ROC) curve (Table 5).

**Table 5:** Model performances

Performance measures	Frequency among models^a^
Numerical outcomes	
R^2^	10
RMSE	9 (7)
NRMSE	4
R value	8
MADP	3
SRD	2
Categorical outcomes	
AUC	9
Accuracy	4
Sensitivity and specificity	3
Hosmer–Lemeshow test	4
NRI	1

The metrics used for the performance evaluation of the models and their frequencies are reported

^a^Between brackets is reported the number of models for which a value of the corresponding metrics is reported

Of the 9 models for which the evaluation was performed with the AUC, the values ranged from 0.73 to 0.97 and 3 models had performances greater than 0.90 [29, 33, 36]. The values of R^2^ ranged from 2.24% [18] up to 77% [22].

### A detailed view of the models

From the above considerations, it emerged that the most used algorithms among models were the regressions, both logistic and linear, whilst the remaining algorithms were almost equally explored. More specifically, by a first broad categorisation of the outcomes based on the ICF model, it was noticeable how logistic regressions were preferred for activities and participation category, whilst the linear regressions for the body functions. For what concerns the relationship among predictor and outcome classes, no preferred choice seemed to be generally taken. Some exceptions are the biomechanical assessment through clinical evaluation class, which was related only to body functions outcomes, and the stroke assessment through laboratory analysis class, interestingly used only for activities and participation category.

**Fig. 5:**
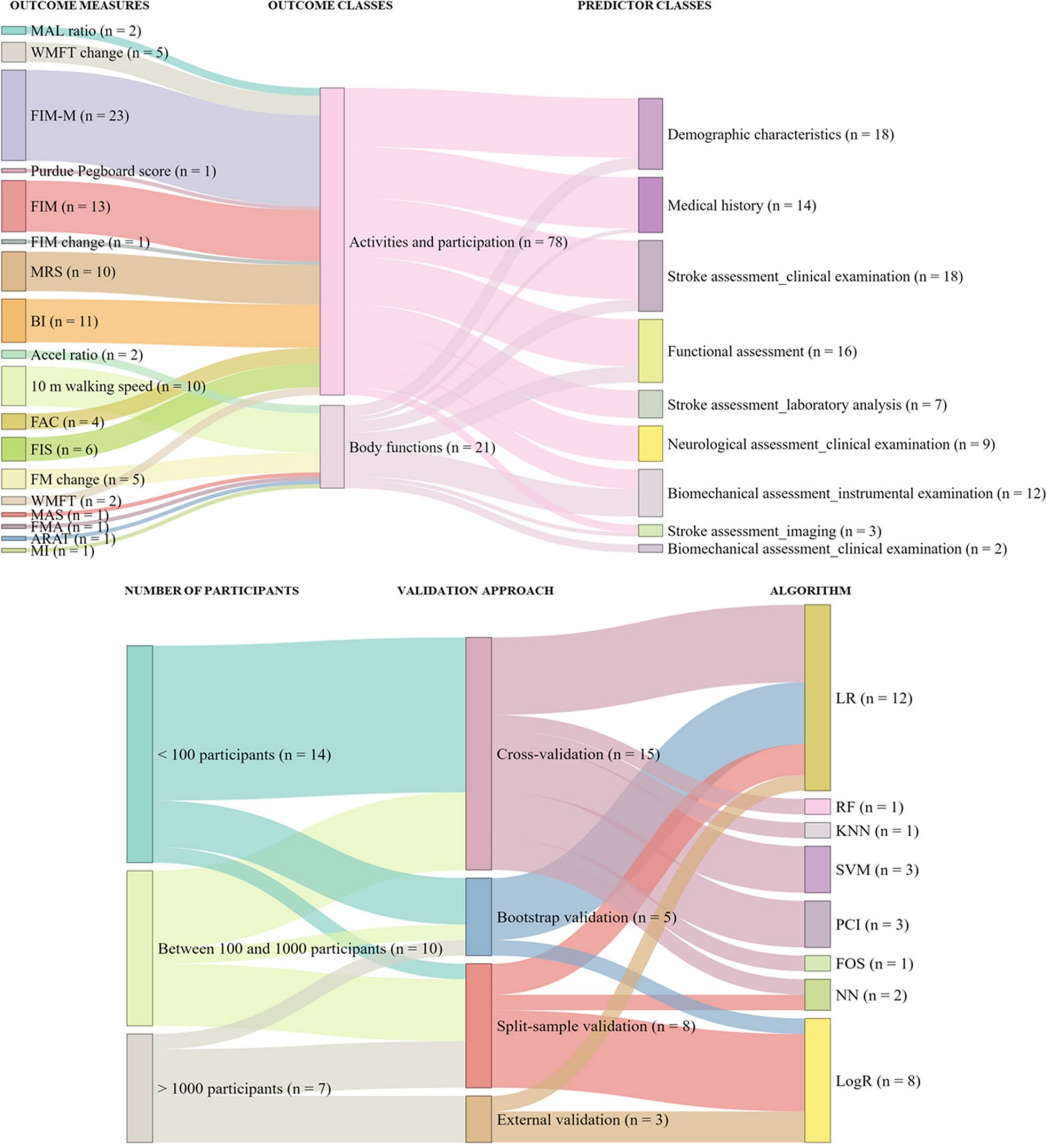
Alluvial charts reporting an overview of the models. They show outcome measures—outcome classes—predictor classes (top) and the number of participants—validation approach—algorithm (bottom)

A global representation of the models investigated in the studies is shown in terms of the outcome measure—outcome classes—predictor classes relationships (Fig. 5, on the top). As mentioned before, for brevity, all the results are displayed considering a categorisation both for predictors and outcomes. Although the predictors are categorised, it summarises the state of the art in terms of models for functional outcome prediction. However, it is not evident any preferred association both in terms of outcome measures with respect to the outcome type and also in the model inputs given a specific outcome. Regarding the model input, almost half of the included studies (8 out of 19) reported among the limitations that the clinical practice drove the choice of features. Indeed, the variables adopted for the models were often obtained from the clinical scales in use in the centre.

At the bottom of the same figure, the number of participants, categorised with the cut-offs of 100 and 1000 patients, is in relation to the validation approach and the algorithms used. From this relation, it is visible that more complex validation approaches, such as bootstrap validation, were used only with linear and logistic regressions. Moreover, the same validation approaches were used with models trained on greater numbers of participants, whilst the cross-validation was performed mostly on models with less than 100 participants. To conclude, no linear relationship between the number of input features and the number of participants was found.

## Discussion

The total number of included studies [19] confirms the interest of the research community in the field of ML tools for stroke prognosis, even though the strict requirements on the validation markedly reduced the number of eligible papers. Indeed, we constrained our analysis to studies including either internal or external validation of the models. In our view, such a requirement is fundamental to assess the generalisation capability and then the real applicability of an ML solution. Limiting the analysis to prognostic factors or ML methods without testing the models on new, unseen data does not allow one to quantify directly the potential of the model without recurring to probabilistic approximations [38]. However, it is worth noting that the exclusion criteria on algorithm validation resulted in a large number of discarded studies, with a number of excluded papers even larger than those discarded for the criteria on the outcome type.

For the description of outcomes and predictors, we decided to report frequencies among models. However, due to the large variability in the number of models reported for each study (from 1 up to 102), we selected those resulting as the best performing on the performance metric reported by the authors. This summarisation was performed among models developed with different algorithms or predictors, while distinct models for each study were presented when different outcome measures or types (categorical or numerical) were used. This process was considered necessary in order to display weighted results among the included studies, without the influence of the number of models that the authors decided to report.

The distinction in classes for both outcomes and predictors was necessary to group the results and generate model comparisons. The generation of classes was performed differently on the outcomes and predictors, as in some cases (5 models out of 31) the input features were given already in categories by the authors. Often, a detailed description of the measures used to define these features was not provided. Hence, in the case of predictors, the categorisation with the ICF model was not possible. Indeed, the proposed predictor categorisation allowed to distinguish among features related to demographics, medical history and clinical, biomechanical and neurological evaluation of stroke and allowed to discriminate among purely clinical, instrumental or laboratory-related features.

Nevertheless, it is important to consider that despite the conciseness and simplicity of this representation, the categorisation of features lacks clinical relevance, a phenomenon that is related to two aspects. First, in the categorisation process, the details on the specific outcome or predictor type were lost. In addition, in the case of outcomes, the categorisation was limited to the measure of the features, neglecting the outcome type, such as motor improvement, functional independence or functional status. Although from the clinical point of view the specific instrument that defines a certain condition has great relevance [39], this aspect needs attention for an appropriate interpretation of the targeted outcome.

For this reason, our suggestion is to detail the specific variables addressed to find elements that can drive the development of new solutions. The application of the PROBAST tool for the analysis of the quality of the included papers highlighted that more than half of the studies were using data from the clinical practice of the specific centre. Hence, the heterogeneity found among models may be explained by a poor standardisation of post-stroke rehabilitation protocols for usual care. Therefore, to fairly compare the performance of ML tools for predictive models and then assess their efficacy for personalised therapies, it would be crucial to establish a common protocol for stroke rehabilitation.

Among the classes of predictors used in the models, the two most populated were demographic characteristics and stroke assessment through clinical examination, not surprisingly used in 18 out of 31 models, as they are related to features that are accessible and fast to collect. Surprisingly, the class of predictors related to biomechanical assessment through instrumental examination was also frequently addressed (12 models), indicating a growing interest in the use of advanced instrumentation for the biomechanical assessment of patients’ kinematics. In particular, the studies from Mostafavi et al. [30] and Bland et al. [22] reported the greatest number of participants over which a biomechanical instrumented examination was performed, with 126 and 269 patients respectively.

Moreover, it was noted that less than half of the papers reporting the feature selection provided the list or the number of the features actually entering the model. Additionally, the PROBAST tool does not fully consider this missing information, considering almost all the models in the predictors section with a low risk of bias. However, a proper description of the feature selection phase is crucial, as it can guarantee not only the reproducibility of the study itself but also the identification of hidden causative associations among outcome and predictors not emerged by classical bio-statistical correlation analyses.

The algorithms most frequently used among the included models were linear and logistic regressions, confirming a preferable choice toward more conventional and interpretable methods, rather than more complex and advanced ones. Going more in detail, a preferred association of logistic regressions and linear regression with outcomes belonging to activities and participation and body functions, respectively, was noticed. This aspect may be addressed as a further confirmation of the need for interpretability of the models. Our findings highlight how outcomes related to higher-level human domains, such as activities and participation outcomes, are rather simplified as categorical features and implemented into logistic regressions.

Another fundamental aspect for the development of reliable predictive models is the sample size. In this review, almost half of the developed models received the answer ‘No’ or ‘Probably no’ in the PROBAST tool question ‘Were there a reasonable number of participants with the outcome?’. The evaluation of this assessment for the PROBAST tool was performed, following instructions available for the tool usage, using the rule of thumb indication of at least 10 samples for each feature. Although this assessment may appear too empirical, the lack of regard for a sufficiently large sample size was confirmed by a non-linear relationship among the number of patients and predictors used. Having larger sample sizes dedicated to the development and validation of the model allows researchers to avoid overfitting complex models and thus to avoid the risk of lacking model generalisation when evaluating new data. Moreover, larger numbers would justify the exploration of more recent ML tools, such as deep learning methods. Among the included studies, those with higher numbers of participants were characterised by multicentric structured databases [20, 21, 24, 26]. Indeed, the implementation of digital infrastructures such as databases, digital clinical folders or data lakes for data storage could promote a digital and data-driven environment, in which a structured and systematic collection of the data is coupled to daily clinical practice.

Differences exist among the possible strategies for method validation; however, we preferred not to further constrain the inclusion criteria. The validation approaches were broadly distinguished among external and internal validation and within the latter type, further groups were created to differentiate among cross-validation, split-sample and bootstrap methods. For what concerns cross-validation, further considerations need to be done, as its use could have a twofold purpose, either for fine-tuning of hyper-parameters or accounting for generalisability, similarly to what is performed with external validation. Especially with complex algorithms, it is important these processes of fine-tuning and generalisability are performed with independent methods, in order to avoid the overfitting of the model on the specific fold configuration. In this study, only four papers [28, 30, 31, 34] clearly reported the final purpose of the validation approach, hence we decided not to perform further categorisations within the group of models validated through cross-validation.

In this work, De Marchis et al. [23], De Ridder et al. [24] and Kӧnig et al. [26] reported both an internal tuning of the parameters and an external validation were used for the development and validation, or calibration, of the model. These studies were among those involving the highest number of participants. Indeed, coherently with the technical characteristics of the approaches, a higher number of participants seemed to be associated with higher complexity of validation approaches (Fig. 5). Although methods like bootstrapping are very efficient and account for sampling variability and cross-validation methods, they should not substitute external validation in prediction research, which should be the best practice. In fact, external validation requires new data to be collected, but it assesses the generalisability of the models by considering changes among populations of patients [40]. For this reason, this effort should usually be planned after model development after a proper tuning of hyper-parameters.

In this review, we found several limitations in the current state of the art: a limited number of participants, high heterogeneity among factors and outcome measures and a small number of models with external validation after appropriate fine-tuning of hyper-parameters. Moreover, the variety of modalities used for the evaluation of the model performance is limiting the possibility to provide a unique, performing model among those found in the literature. Despite these methodological restrictions, the results show it is possible to identify the most frequently used predictors and algorithms given a specific outcome; this ability provides insight into the state of the art and a useful perspective for the development of new solutions (Fig. 5).

## Conclusions

Predictive models can be a very promising support tool for clinicians. ML algorithms can be easily deployed for this purpose, due to their capability of handling large cohorts and high dimensional datasets; indeed, once trained, they provide accurate estimates at a low cost. Among the advantages, this kind of solution could stimulate a more data-driven approach in clinical practice, promote a more structured definition of studies and reduce the gap between clinical and research areas. For this reason, we suggest promoting additional research in this field, with larger datasets, external validation of the models and an accurate and scientifically driven selection of outcomes and predictors. Furthermore, the implementation of defined protocols and registers for the evaluation of post-stroke patients in clinical practice is strongly suggested.

This would allow for larger datasets and a broad variety of features, including instrumental ones, that are crucial elements in the development of predictive models. We are convinced that to optimise and personalise the rehabilitation treatment, future research should lead to extensively validated ML methods that become embedded in decision support tools of daily use.

## Supplementary Information

**Additional file 1. Table 5**. Extended data extraction.

## Data Availability

All data generated or analysed during this study are included in this published article and its Additional files.

## Outcome measures

ARAT: Action research arm test
BI: Barthel Index
FAC: Functional ambulation categories
FIM: Functional independence measure
FIM-M: Functional independence measure-motor
FIS: Fatigue impact scale
FM: Fugl-Meyer
FMA: Fugl-Meyer assessment
MAL: Motor activity log
MAS: Modified Ashworth Scale
MI: Motricity Index
MRS: Modified Rankin Scale
SDMT: Symbol digit modalities test
WMFT: Wolf motor function test

## Algorithms

ANN: Artificial neural networks
FOS: Fast orthogonal search
kNN: K-nearest neighbours
LR: Linear regression
LogR: Logistic regression
PCI: Parallel cascade identification
RF: Random forest
SVM: Support vector machine

## Performance measures

AUC: Area under the curve
MADP: Mean absolute deviation percentage
NRI: Net Reclassification Index
NRMSE: Normalized root mean square error
RMSE: Root mean square error
SRD: Smallest real difference
